# Advanced Carbon-Based Polymeric Nanocomposites for Forensic Analysis

**DOI:** 10.3390/polym14173598

**Published:** 2022-08-31

**Authors:** Ana M. Díez-Pascual, Daniel Lechuga Cruz, Alba Lomas Redondo

**Affiliations:** 1 Universidad de Alcalá, Facultad de Ciencias, Departamento de Química Analítica, Química Física e Ingeniería Química, Ctra. Madrid-Barcelona Km. 33.6, 28805 Alcalá de Henares, Madrid, Spain; 2 Departamento de Química Inorgánica y Orgánica, Facultad de Ciencias Experimentales, Universidad de Jaén, 23071 Jaén, Spain; 3 Universidad de Alcalá, Departamento de Teoría de la Señal y Comunicaciones, Ctra. Madrid-Barcelona Km. 33.6, 28805 Alcalá de Henares, Madrid, Spain

**Keywords:** carbon nanomaterials, forensic science, sensor, polymer nanocomposites

## Abstract

Nanotechnology is a powerful tool and fast-growing research area in many novel arenas, ranging from biomedicine to engineering and energy storage. Nanotechnology has great potential to make a significant positive contribution in forensic science, which deals with the identification and investigation of crimes, finding relationships between pieces of evidence and perpetrators. Nano-forensics is related to the development of nanosensors for crime investigations and inspection of terrorist activity by analyzing the presence of illicit drugs, explosives, toxic gases, biological agents, and so forth. In this regard, carbon nanomaterials have huge potential for next-generation nanosensors due to their outstanding properties, including strength combined with flexibility, large specific surface area, high electrical conductivity, and little noise. Moreover, their combination with polymers can provide nanocomposites with novel and enhanced performance owed to synergy between the composite components. This review concisely recapitulates up-to-date advances in the development of polymer composites incorporating carbon-based nanomaterials for forensic science. The properties of the different carbon nanomaterials, several methods used to analyze functional polymeric nanocomposites, and their applications in forensic investigation are discussed. Furthermore, present challenges and forthcoming outlooks on the design of new polymer/carbon nanomaterial composites for crime prevention are highlighted.

## 1. Introduction

Nanotechnology is the convergence of the traditional fields of chemistry, physics, and biology to synthesize, make, design, manipulate, and study matter on the nanometer scale. Numerous areas, including biomedicine, physics, material sciences, energy storage, and electronic engineering, use nanomaterials for different objectives [1]. Nanotechnology has great potential to contribute significantly to forensic science [2,3], which deals with the identification and investigation of crimes, finding relationships between pieces of evidence and perpetrators. It usually analyzes the evidence found at the crime scene (i.e., hair, DNA, protein, nails, paints, fibers, minerals, plastics, etc.) using certain techniques and methods. Nanomaterials in forensic science make the investigation procedure shorter, more accurate, sensitive, and selective. For instance, nanomaterials offer new solutions to gather and identify DNA from fingerprints, explosives, and gunshot residue (GSR) [4], amongst others. Inorganic nanoparticles such as Au or Ag, metal oxides such as TiO_2_ and ZnO, semiconductors such as silicon and ceramics, and carbon-based nanomaterials such as fullerenes (C_60_), carbon nanotubes (CNTs), carbon nanofibers (CNFs), carbon black (CB), graphene (G), and its related forms, namely graphene oxide (GO) and reduced graphene oxide (rGO), are leading examples of nanomaterials [5,6,7].

Nanomaterials, especially those derived from carbon, have exceptional physical and chemical properties due to their large surface area and dimensions on the nanoscale, combined with their superior strength, flexibility, and electrical conductivity. Their optical properties and reactivity are influenced by their size, shape, and structure [8]. Their surface functionalization capability enables interaction with a lot of compounds. Thus, polymers, artificial ligands, and biomolecules can interact with nanomaterials to modify them for their desired uses [9]. Furthermore, functionalization has been demonstrated to prevent nanomaterial agglomeration, leading to improved physical, chemical, and mechanical characteristics of the resulting composites due to synergy [10,11].

A polymer nanocomposite is a composite material composed of nanomaterials bound together by a matrix of organic polymer. Many commercially available polymers have been used as matrices of nanocomposites. Amongst the most widely used, especially for sensing devices, are conductive polymers such as polypyrrol (PPy) and polyaniline (PANI) and biocompatible polymers such as polyethylene glycol (PEG) and chitosan (Chit) [12,13,14]. The idea is to form a huge interface between the nanomaterial and the chains of the neat polymer, which is expected to result in extraordinary properties compared to conventional microfilled polymer composites. 

This paper deals with the role of these polymer/nanomaterial composites in the field of forensic science, with a special focus on those incorporating carbon-based nanomaterials. The applications of polymers and nanostructures in forensics range from the detection of drugs, toxins, explosives, toxic gases, etc. to nanosensors and fingerprinting technology. Many microscopic, chromatographic, and spectroscopic techniques have been applied to identify residues and signs in the crime scene at the nanometer range. The classification of carbon nanomaterials, the most common methods applied to characterize nanomaterials and polymers, and the most important applications of these functional polymer/nanomaterial composites in forensic research are described. 

## 2. Carbon-Based Nanomaterials

**Figure 1:**
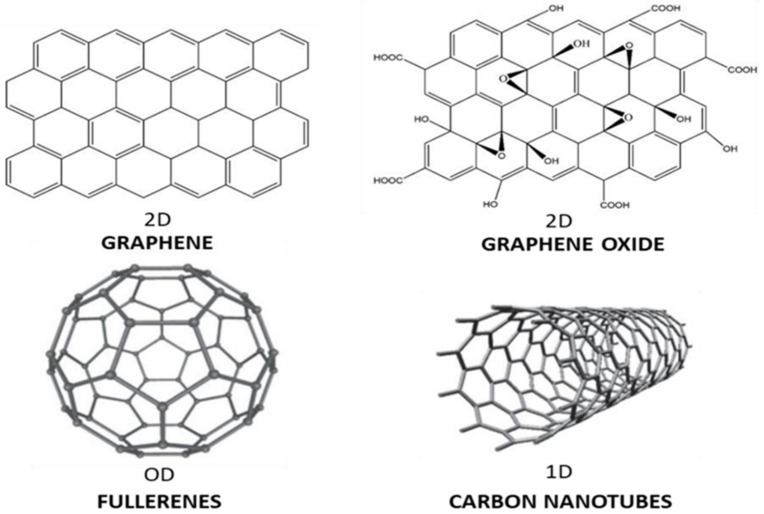
Schematic representation of carbon-based nanomaterials: 0D fullerenes, 1D carbon nanotubes (CNTs), 2D graphene (G), and graphene oxide (GO).

Their major component is carbon. Based on electron movement, they can be classified as 0D, 1D, 2D, or 3D [15], as displayed in Figure 1. 0D nanomaterials are those that have dimensions that are smaller than 100 nm, such as fullerenes and carbon quantum dots (CQDs). 1D nanomaterials have 2 dimensions smaller than 100 nm such as carbon nanotubes (CNTs), 2D nanomaterials only have 1 dimension on the nanoscale such as graphene and grapheme oxide, and 3D nanomaterials have dimensions that are larger than 100 nm but are composed of smaller entities.

### 2.1. Fullerenes

Fullerenes are spherical or ellipsoid in shape, with a hollow structure, and hold sp^2^ and sp^3^ hybridized carbon atoms. They comprise bonded rings of five to seven atoms. Fullerenes were discovered in 1985 by Kroto and Smalley, working at Sussex University, in the sooty residue generated via vaporization of carbon in a helium atmosphere [16]. The mass spectrum of the residue showed peaks corresponding to ball-like polyhedral molecules, which they named “buckyballs”. Since they had an exact mass of sixty or seventy, they were called C_60_ and C_70_. The most well-known molecule is C_60_, named Buckminster fullerene [17]. It consists of a truncated icosahedron, bearing a resemblance to a football composed of 20 hexagons and 12 pentagons.

Their structure is distinctive since it is borderless, uncharged, and lacks boundaries or unpaired electrons. These attributes differentiate fullerenes from other carbon allotropes such as graphite or diamond, which have electrical charges and edges with dangling bonds. Fullerenes are soluble in common organic solvents, such as toluene, chlorobenzene, and 1,2,3-trichloropropane, at room temperature [18]. They are chemically reactive and can be combined with polymers to form composites with novel physical and mechanical properties. They are strong antioxidant agents, have sensing/detection capabilities and antimicrobial activity, can easily penetrate cell membranes, and are frequently applied to develop gene/drug delivery systems [19].

### 2.2. Quantum Dots

**Figure 2:**
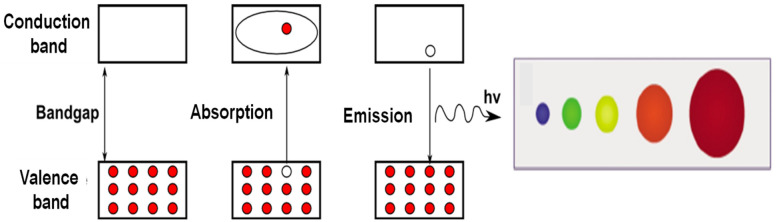
Representation of the confinement effect in QDs and the emission of photoluminescence. The color depends on the QD size.

Quantum dots (QDs) are 0D semiconducting NPs with different properties from micro- and macro-particles due to the confinement of the electrons in all directions [20]. When QDs are irradiated with UV light, an electron is excited from the valence to the conduction band, and when it returns to the ground state, it emits electromagnetic radiation; this process is known as “photoluminescence” (Figure 2). Their optoelectronic properties depend on both their size and shape. Smaller QDs emit shorter wavelengths and hence are blue and green while larger ones are red or orange.

QDs can be synthesized via numerous methods, typically top-down and bottom-up approaches [21]. Examples of the former are molecular beam epitaxy and X-ray lithography. Bottom-up approaches include colloidal synthesis, self-assembly, and electrical gating. The most simple, economic, and versatile is colloidal synthesis, in which precursors are heated at high temperatures to decompose and generate monomers that subsequently nucleate and generate nanocrystals. The concentration of monomers and growth temperature are critical factors that determine the crystal size [22]. Plasma synthesis is another approach used to produce QDs, especially those with covalent bonds. Additionally, hydrothermal synthesis and photo-Fenton reaction techniques can be employed.

These nanomaterials have an elevated extinction coefficient and fluorescence quantum yield combined with good photobleaching stability. They have potential applications in many fields, including diode lasers, solar cells, LEDs, inkjet printings, electron transistors, amplifiers and biological sensors, microscopy, and medical imaging [22,23]. They can be used as donor fluorophores in Föster resonance energy transfer. Moreover, their improved photostability allows for the development of highly sensitive devices for cellular imaging, enabling the acquisition of high-resolution 3D images. They can be employed for tumor targeting under in vivo conditions [24]. They also possess antibacterial properties towards Gram-positive and Gram-negative bacteria [25].

### 2.3. Carbon Nanotubes

CNTs are 1D, rolled-up layers of carbon atoms with sp^2^ hybridization (Figure 1) [26]. They can be divided into single-walled carbon nanotubes (SWCNTs) and multi-walled carbon nanotubes (MWCNTs. They are lightweight and have remarkable mechanical, thermal, and electrical properties, which depend on their diameter, length, and chirality [27]. Their stiffness is the highest amongst any known material, with a modulus close to 1000 GPa [28]. They can be conducting, semiconducting, or insulating. The conducting ones have a current density in the order of 4 × 10^9^ A/cm^2^, which is much higher than that of metals such as Ag (10^5^ A/cm^2^). They also show very high thermal conductivity (more than 10^3^-fold that of metals such as Cu) and display very high thermal stability, up to 700 °C under air atmosphere and 2800 °C under vacuum [29]. Although, they tend to aggregate, which leads to property deterioration. Henceforth, functionalization with polymers [30] or other molecules is frequently required.

### 2.4. Graphene and Its Derivatives

Graphene (G) is a 2D atomically thick carbon nanomaterial comprising a honeycomb lattice of sp^2^ carbon atoms. It was first prepared eighteen years ago during exfoliation of a graphite pencil with Scotch tape [31]. G has outstanding properties, together with elasticity and lightness. In particular, it has very high thermal conductivity [32], about 10-fold higher than that of other metals such as Cu, and exceptional electrical conduction (up to 5000 S cm^−1^). Moreover, it is one the toughest compounds on Earth and significantly stiffer than steel [33]. These exceptional properties make G a perfect candidate for many applications such as fuel cells, sensors, nanocomposites, photovoltaic and flexible electronic devices, etc. [12,34,35].

G can be modified with other molecules (or macromolecules) by two main strategies, namely non-covalent and covalent, to form functional nanocomposites. Covalent interactions occur via the formation of chemical bonds through approaches named “grafting-from” and “grafting-to” [7]. Conversely, the non-covalent approach consists of the adsorption of polymers or small molecules onto G through physical interactions such as Van der Waals, hydrogen bonding, π-π, cation-π, etc. [36,37].

**Figure 3:**
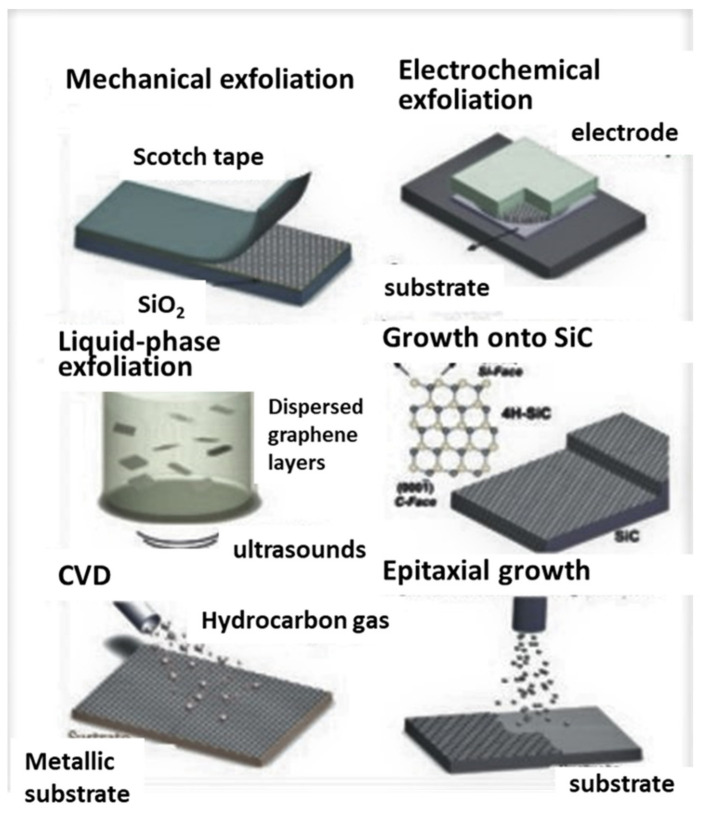
Most typical techniques for graphene synthesis.

G synthesis is typically performed in two ways, namely bottom-up and top-down approaches. In the first methods, graphite is the starting compound, which can be exfoliated mechanically (scotch tape method), in the liquid phase (typically with the aid of ultrasound to disperse the graphene layers [38,39]) or electrochemically, by applying a potential, as depicted in Figure 3 [40,41].

The bottom-up techniques rely on G assembly from molecular precursors. One of the most employed is epitaxial growth, although it is one of the most expensive methods since it requires a SiC substrate that is heated at very high temperatures. However, it enables precise control over the film thickness via tailoring of the process parameters. Another widely used method is chemical vapor deposition (CVD), an economic and large-scale method used to yield high-quality graphene [42]. Hydrocarbon gas is saturated at very high temperatures onto a metallic substrate. When it cools down, the solubility of carbon decreases, and the graphene film is produced.

On the other hand, graphene derivatives are currently used for numerous applications, including the fabrication of biosensors. Amongst them, the most important is graphene oxide (GO), an oxidated arrangement of G with oxygenated functional groups, typically synthesized via Hummer’s method using oxidants [43]. Another well-known derivative is reduced graphene oxide (rGO), which is prepared by reducing GO via a chemical treatment or elevated heat [44] using synthetic reducing agents such as hydrazine or sodium borohydride, or more recently, sustainable reductants such as ascorbic acid [45].

## 3. Forensic Analysis of Polymer/Carbon Nanomaterial Composites

The analysis of polymer/nanomaterial nanocomposites is currently performed using a huge number of techniques, generally classified into three types: microscopic, spectroscopic, and chromatographic.

### 3.1. Microscopic Techniques

**Figure 4:**
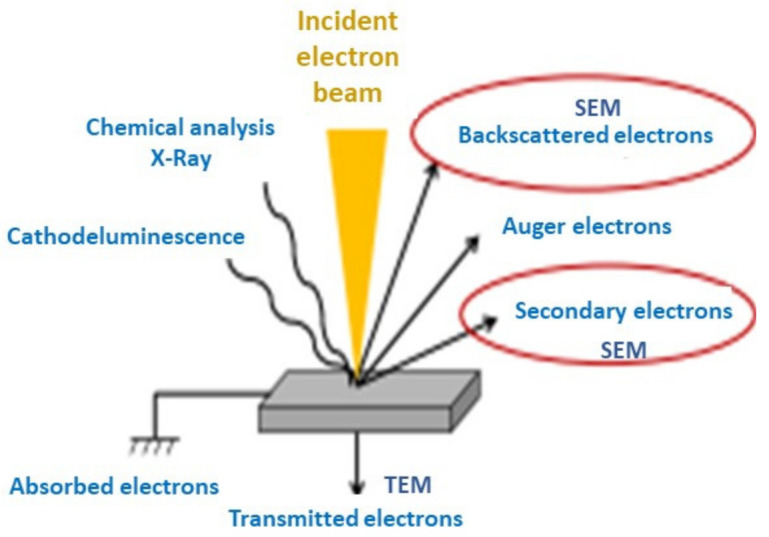
Different types of signals generated in SEM and TEM analysis. The most important are circled in red color.

Optical and electron microscopy techniques such as atomic force microscopy (AFM), transmission electron microscopy (TEM), and scanning electron microscopy (SEM), provide information on the structure, form, dimension, and surface morphology [46]. They are frequently used in forensics for evidence tracking. In SEM analysis, the sample surface is scanned with a beam of electrons, and secondary electrons of low energy are generated that provide topographical information of the most external layers of the sample (Figure 4). In addition, backscattered electrons are produced, the intensity of which is directly related to the atomic mass of the sample elements, hence providing information about its chemical composition. It is a very versatile technique, with an average resolution of 5 nm. However, non-conducting samples need to be covered with a metallic overlayer to avoid charging during electron irradiation [47].

Typically, SEM analysis is coupled to an energy dispersive X-ray spectroscopy (EDXS) detector, which also permits information about the sample composition. It is a semiquantitative analysis since it yields the amount (in percentage) of each sample element.

TEM uses a beam of electrons accelerated by a high voltage as the source, which penetrates across the sample [48]. It provides a higher resolution than SEM (i.e., 0.2 nm), but the sample preparation is more tedious and requires very thin samples. For such a purpose, samples are first fixed with glutaraldehyde, typically embedded in a resin matrix, and then cut with an ultramicrotome into very fine slices. It provides evidence about the size and presence of structural defects.

AFM is the most common technique in forensics for the analysis of polymeric nanocomposites since it enables surface monitoring at the nanoscale. It uses a microcantilever to inspect the sample surface. The cantilever deflection is recorded and converted into a 3D image. AFM has a higher resolution than SEM and TEM and does not require sample preparation. AFM is widely applied in various forensic fields to analyze firearms, explosives, hair, blood, and documents to examine the crossing over of two different inks [49]. It is very versatile since it allows investigation of solid, liquid, and gas samples.

### 3.2. Spectroscopic Techniques

These methods rely on the interaction of the incident radiation with matter. Dynamic light scattering (DLS) is applied to investigate the size of nanomaterials in liquid medium [50]. It offers insight about the hydrodynamic radius; hence, the size differs from that obtained in the solid state by SEM and TEM.

Infrared (IR) spectroscopy usually employs Fourier transform (FTIR) instrumentation. When the sample molecules absorb IR light, they are excited from the ground vibrational state to an excited state, and this transition provides information about the functional groups present on its surface [51,52]. It is widely used to characterize carbon nanomaterials. It is a versatile, simple, and non-destructive technique performed under ambient conditions.

Raman spectroscopy also gives information about the functional groups on the sample surface, such as IR spectroscopy. While IR measures the modification in the dipole moment of the molecule, Raman is based on the modification in its polarizability [53]. Raman is a nondestructive technique that can be combined with microscopy to obtain 3D images.

Another spectroscopic technique is UV-visible spectroscopy, which offers qualitative information on the samples in solution by showing the peak shift and broadening and changes in the spectral intensity. Solid-state nuclear magnetic resonance (NMR) offers insight about the conformation and structure of molecules and their interactions with other surrounding molecules [54], and it is frequently applied to analyze NPs. In this technique, nuclei are perturbed under a strong constant magnetic field and react by generating an electromagnetic signal related to the field experienced by the nucleus in the radiofrequency region [54].

### 3.3. Chromatographic Techniques

Separation, detection, and characterization of polymer nanocomposites can be performed by different chromatography techniques, which involve mass transfer between two phases, one mobile and the other stationary. Depending on the stationary phase, it can be liquid chromatography or gas chromatography. Chromatographic techniques are typically coupled with mass spectrometry (MS), which generates multiple ions from the sample being investigated and then separates them according to their mass-to-charge (*m/z*) ratio. High-performance liquid chromatography with MS detection (HPLC/MS) is a widely used technique to detect illegal drugs. Gas chromatography-mass spectroscopy (GC/MS) has been broadly applied to analyze hair, plasma, blood, drugs, etc. [55].

## 4. Applications of Polymer/Carbon Nanomaterials in Forensics

### 4.1. Detection of Illicit Drugs

Forensic analysis of drugs typically involves initial colorimetric tests (i.e., Simon, Marquis, Zimmermann, Duquenois-Levine, Scott) and then confirmatory tests by various chromatography techniques such as HPLC, GC/MS, FTIR, Raman spectroscopy, etc. The combination of nanomaterials with these techniques allows a more efficient, sensitive, and sensible approach [56].

GO has been used for the detection of illegal drugs since it can act as a fluorescence quencher of aptamers. Although, there are certain related problems, since quenching involves the physical adsorption of the aptamer onto GO, which limits the sensitivity of the detection and can result in false signals. Poly-cytosine (poly-C) DNA, sequences of DNA comprising cytosines in the range of 0–30, can be strongly adsorbed on many common nanomaterials, and this approach can be used to further enhance the sensitivity in the analysis of illegal formulations. In this regard, Shi et al. [57] used an aptamer with poly-C adsorbed on the basal plane of GO to identify cocaine. For further property improvement and to avoid the binding of other drugs, a nonionic surfactant (Tween 20) was used, which is prone to interaction with GO via its lipophilic chains. Using this tactic, the limit of detection (LOD) was decreased to 2.45 pM. The results show that our method is a promising technique for analytical application.

**Figure 5:**
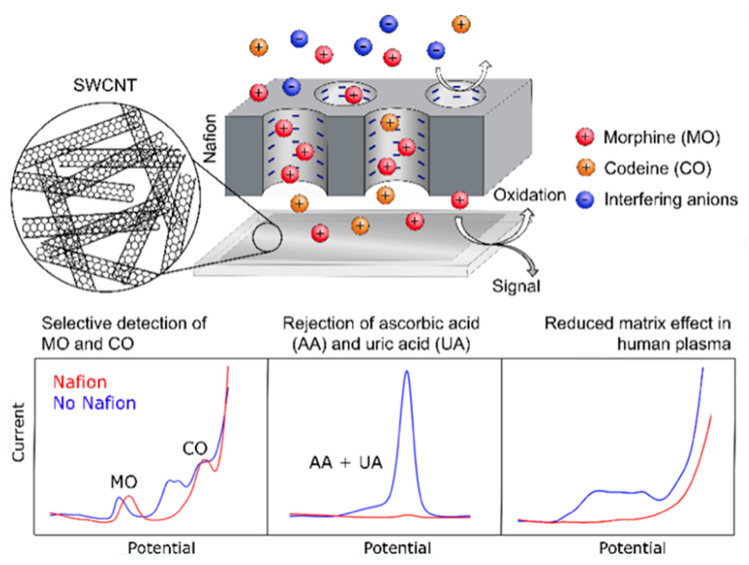
**Top**: Representation of the electrode comprising Nafion and SWCNT for selective detection of morphine and codeine. **Down**: Cyclic voltammograms (I vs. V) for the neat SWCNT electrode (blue) and Nafion-coated SWCNT electrode (red). Reprinted from [59], copyright 2019, American Chemical Society.

Nafion is a perfluorosulfonic acid (PFSA)/polytetrafluoroethylene (PTFE) copolymer that is broadly used in electrochemistry [58]. Wester et al. [59] developed an electrochemical sensor comprising a Nafion membrane and a network made of SWCNTs to selectively detect morphine (MO) and codeine (CO). SWCNT network electrodes were manufactured via a dry displacement technique and dip-coated with the Nafion membrane. The effectiveness of the sensor was confirmed by the successful concurrent detection of MO and CO in a solution with a large excess of ascorbic acid (AA) and uric acid (UA, Figure 5). The Nafion membrane comprises nanoscale-linked channels coated by sulfonic groups, which are negatively charged. Therefore, only cationic molecules such as opioids can penetrate it. This is the main advantage of this type of sensor when dealing with biological media, given that most of the interferents at neutral pH are negatively charged, such as AA and UA. Thus, the Nafion-coated SWCNT electrode showed superior selectivity and sensitivity for analyte sensing in multicomponent biological media, and was able to detect MO in the range of 0.05–10 μM and CO in the range of 0.1–50 μM. This promising electrode also allowed measurements in unprocessed (just slightly diluted) human plasma without protein precipitation.

The same group used the Nafion-coated SWCNT electrode to investigate the electrochemical performance of a strong opioid commonly employed as a palliative (oxycodone), and its derivatives, namely noroxycodone and oxymorphone [60]. The electrode was selective for oxycodone when both metabolites were present, and was able to quantify this opioid in buffer solution in the concentration range of 0.5–10 μM, with a low LOD of 85 nM. This work corroborates that the Nafion-coated electrode has great potential to be used in forensic biology for real measurements.

Ansari et al. [61] developed a novel sensor to detect oxycodone based on a molecularly imprinted polymer (MIP) comprising GO and carbon dot nanoparticles (CD NPs). The sensor was fabricated via ultrasonication-aided microextraction and HPLC. The surface morphology, adsorption capacity (AC), surface functional groups, thermal stability, and magnetic properties of the synthesized CD NPs were investigated via SEM, BET, FTIR, TGA, and VSM, respectively. The adsorption isotherms of the modified MIPs obeyed the Langmuir equation, and the highest AC and the imprinting factor were close to 100 mg/g and 3.4, respectively. For the best conditions, which were attained by modeling (i.e., response surface methodology), the linear interval ranged between 0.001 and 2 µg/mL, with an LOD of 0.0008 ng/mL. The mean recoveries of drug in human urine samples were found to be in the range of 92.50 to 103.20%, with relative standard deviations smaller than 3.65%.

**Figure 6:**
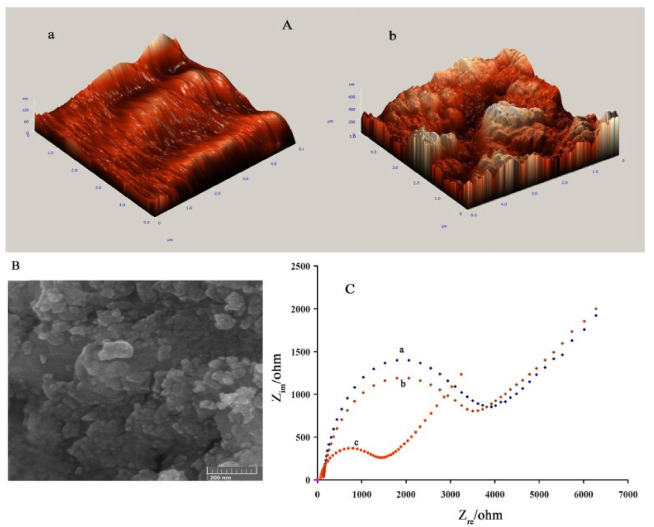
(**A**) AFM images of neat PGE (**a**) and PGE/CuO-NPs/PPy nanocomposite (**b**). (**B**) SEM micrograph of the nanocomposite (**C**) Nyquist plots of neat PGE (a), PGE/PPy (b), and the ternary nanocomposite (c). Reprinted from [65], copyright 2020, Elsevier.

Tramadol (C_16_H_25_NO_2_) is an alkaloid that alleviates pain via hindering the uptake of serotonin and norepinephrine [62]. This powerful palliative is broadly applied for postoperative treatments worldwide [63]. Although this alkaloid is regarded as a safe medicine, an overdose can lead to severe effects and even cause death [64]. Arabali et al. designed a tramadol electrochemical sensor based on a pencil graphite electrode (PGE) modified with PPy and CuO nanoparticles. The morphology of the developed sensor was investigated via AFM and SEM images (Figure 6). The electrocatalytic activity of PGE/CuO-NPs/Ppy for tramadol oxidation determination via the square wave voltametric (SWV) technique was outstanding, with a 4.6-fold rise in the oxidation current related to the bare PGE electrode. The interval of linearity was 0.38–5.0 nM, and the LOD was around 1.0 nM. Furthermore, the developed electrode was successfully applied to drug samples for tramadol detection [65].

Analogously, Bagheri et al. used a CPE with silver-decorated graphene and MIP in a sensor for tramadol-selective detection. The authors decorated G layers with AgNPs using sodium borohydride as the reducing agent. MIPs were synthesized via polymerization of a monomer in a medium comprising the analyte. The sensor was able to accurately detect tramadol in pharmaceutical medicines, with an LOD of 2.0 nM and lineal interval ranging from 3.50 × 10^−9^ to 1.00 × 10^−2^ M [66].

Ketamine is a widely applied sedating drug that causes numerous side effects, including amnesia and painlessness. Although, significant issues regarding illicit use of this soporific drug to spike alcoholic drinks above the allowable dosage have arisen. Narang et al. [67] developed an ultra-fast sensitive technology for the electroanalytical detection of ketamine based on an electrochemical fluidic paper-based analytical device (EμPAD). A paper chip was designed with zeolite flakes and GO nanocrystals. The device showed a broad interval of linearity, from 1 to 0.005 nM, with a very low LOD of 0.001 nM. This EμPAD is considerably less expensive than conventional metal-based electrodes, requires only microlites of sample, and shows an improved performance, including a reduced LOD and minimal response time.

MIPs have also been applied in G-based sensors for selective functionalization of ketamine. Recently, Fu et al. [68] designed an MIP membrane on a metal–organic framework (MOF)-modified G sheet to be used as an electrode in an electrochemical sensor in order to quantity the concentration of ketamine in corporal fluids. Firstly, MOF was synthesized via a solvothermal reaction using 1,4-benzendicarboxylic acid and metallic zirconium. G and Zr-MOF were mixed in ethanol and deposited on the top of the sensor. Separately, monomers were mixed with ketamine and the blend was deposited onto the electrode, forming a thin MIP layer. Cyclic voltammetry (CV) was applied to characterize the electrode, which displayed a selective response to ketamine and its metabolites, while it did not detect methamphetamine derivatives, dopamine (DOP), or AA. Ketamine was accurately measured in urine and saliva, with an LOD of 0.04 nM and a broad interval of linearity, from 0.1 nM to 0.4 M.

Saisahas et al. [69] prepared a simple and inexpensive graphene-based sensor as a methamphetamine sensor via a one-step solvent free fabrication method. For this purpose, Kapton tape was placed on a polyethylene terephthalate (PET) substrate and then a CO_2_ laser was used to grow G on its surface. The flexible sensor was tested in a cyclic voltammetry system, leading to an LOD of 0.3 g/mL. To assess its selectivity, the sensor response in the presence of UA, AA, urea, and sugar was recorded, and it showed high selectivity towards methamphetamine. The sensor was tested with real samples such as saliva and samples recuperated from plastic and glass surfaces, and very high recoveries were attained, indicating its suitability for criminal studies.

**Figure 7:**
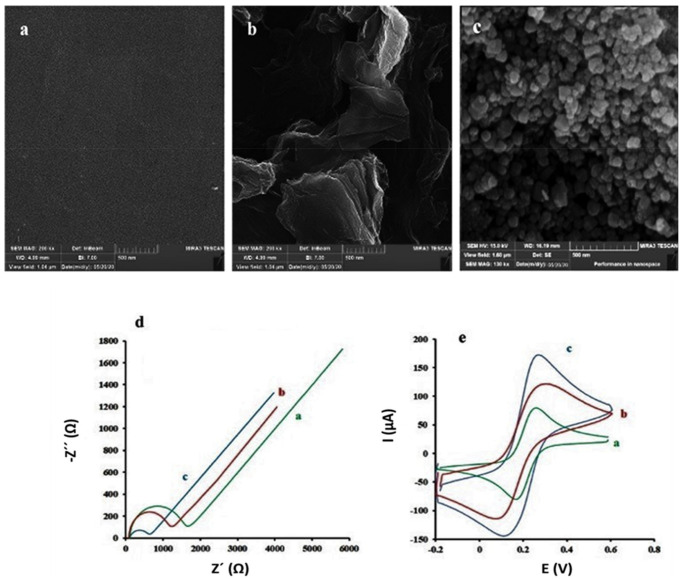
SEM micrographs of neat GCE (**a**), rGO/GCE (**b**), and rGO/GCE/Fe_3_O_4-_PPy (**c**). Nyquist plots (**d**) and CV (**e**) of neat GC (a), rGO/GC (b) and rGO/GCE/Fe_3_O_4-_PPy (c). Taken from [70], copyright 2021, Elsevier.

Another sensor for sensitive methamphetamine detection was designed by Riahifar et al. [70], who used functionalized rGO. First, GO was reduced on the surface of a GCE. Then, FeCl_3_ was converted into Fe_3_O_4_ using NH_4_OH, and pyrrol monomers were electropolymerized, leading to a polypyrrol shell wrapping the Fe_3_O_4_ core. The core-shell NPs were added to rGO and dried at room temperature. Typical SEM micrographs of neat GCE, rGO/GCE, and rGO/GCE/Fe_3_O_4-_PPy are shown in Figure 7. Urine and human blood serum were tested, and the Nyquist plots and cyclic voltagrams for their detection are also shown in Figure 7. The LOD was about 0.01 µM, and the linear interval was 0.0005–0.2 mM. Although the selectivity of this method was not explored, no response to salts or amino acids was recorded.

**Figure 8:**
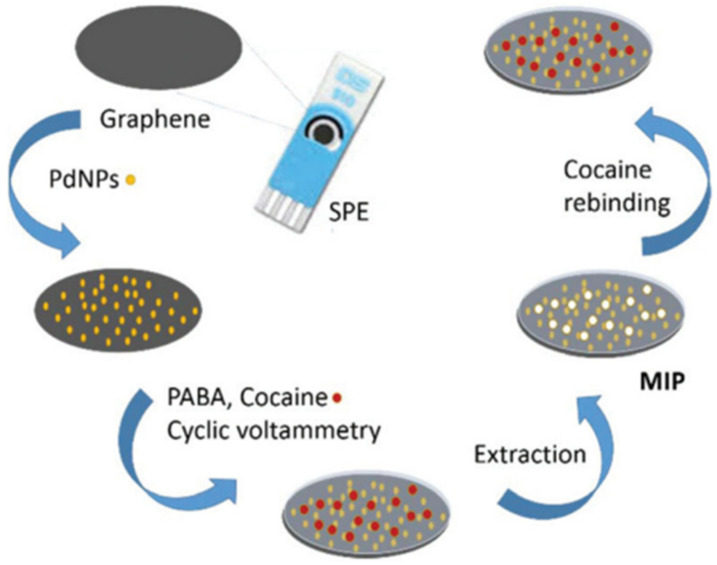
Synthesis of a sensor for cocaine quantification via MIP electropolymerization onto electrodes with graphene-modified with Pd-NPs. Taken from [71], copyright 2019, Royal Society of Chemistry.

Cocaine is amongst the most marketed illegal drugs worldwide. Hence, quick and sensitive approaches for the identification and quantification of this drug are highly desirable. Florea et al. [71] prepared an electrode for selective detection of cocaine via MIP assembly on the surface of Pd-decorated G. MIP was synthesized by the CV technique using the drug and aminobenzoic acid as a template and a monomer, respectively, as depicted in Figure 8. Experimental parameters associated with the deposition of Pd NPs such as pH, composition of the electropolymerization mixture, and so forth were investigated and tailored. The SWV technique was applied to measure the drug amount, with an interval of linearity in the range of 0.1–0.5 M and an LOD of 0.05 M. Real samples of this drug, saliva, and river water (drug spiked) were analyzed, and the electrode could quantify cocaine amounts near the added levels.

Muñoz et al. [72] designed a polylactic acid (PLA)/G electrode to quantify small amounts of cocaine using SWV. An LOD of 6 µM was attained, with a linear concentration range of 20 and 100 μM, and without the interference of frequent adulterants of confiscated drugs such as caffeine, phenacetin, paracetamol, etc. Despite presenting a new application and the inexpensiveness of the developed cocaine sensor, this electrode and the corresponding approach were first reported by another group [73], who applied it to quantify phenolic compounds such as DOP.

Hashemi et al. [74] designed a new label-free aptasensor comprising a screen-printed carbon electrode (SPCE) modified with 3D magnetic rGO/PANI/AuNPs for cocaine detectioni. A drug aptamer with thiol groups was fixed onto the rGO/PANI/AuNPs nanocomposite. The aptasensor mechanism relied on the rise in the resistance to electron transfer of iron hexacyanide in the presence of the analyte. The novel electrochemical cell configuration was sustainable, reusable, lead to repeatable responses, and reduced the electrolyte volume. The nanocomposite and the modified electrode were characterized via CV, EIS, SEM, and FTIR. After optimization of the method, the drug was measured in the range of 90–8500 µM, with an LOD of 30 µM. The designed sensor was useful to quantify this drug in urine and serum, leading to good values.

Xylazine, a phenothiazine derivative, named as “tranq”, is a strong sedative that was first synthesized for hypertension treatment, but it causes bradycardia and temporary hyperglycemia. Due to its secondary effects, the Food and Drug Administration restricted its use [75]. In the last years, xylazine has become a widespread entertaining drug around the world and has been commonly used to adulterate illegal drugs such as heroin and cocaine and mixtures of them [76]. However, the toxicity of this drug together with others remains unknown due to the restriction of its administration to humans [77]. With the aim of detecting xylazine, Saisahas et al. [78] prepared a electrochemical device that uses paper as a substrate. Their inexpensiveness, lightness, flexibility, portability, sustainability, and appropriateness for mass manufacture make them highly valuable in forensics. G ink was deposited on the paper and functionalized with PANI, offering a big surface area that promoted the transfer of electrons. The π-π interactions amongst the benzenic rings of G and those of xylazine augmented the analyte adsorption. Differential pulse voltammetry (DPV) was used to test this sensor directly on xylazine, and a linear response was found from 0.18 to 5 μg mL^−1^ and from 5 to 100 μg mL^−1^, with an LOD of 0.06 μg mL^−1^. The sensor was applied to detect this drug in spiked drink samples, and the recoveries ranged from 84 to 106%. A portable system can be designed to detect illicit drugs in forensic investigations

### 4.2. Sensors for Doping Substances

The usage of illegal banned compounds for improvement of athletic ability, preparation, and operation is recognized as “doping”. For years, experts and elite sportspersons have extensively used substances to boost their sport activities. The most frequent types of doping drugs comprise not only illegitimate materials such as amphetamine, cocaine, ephedrine, or narcotic analgesic but also compounds sold as nutritious complements such as anabolic steroids, peptide hormones, and diuretics, among others [79].

**Figure 9:**
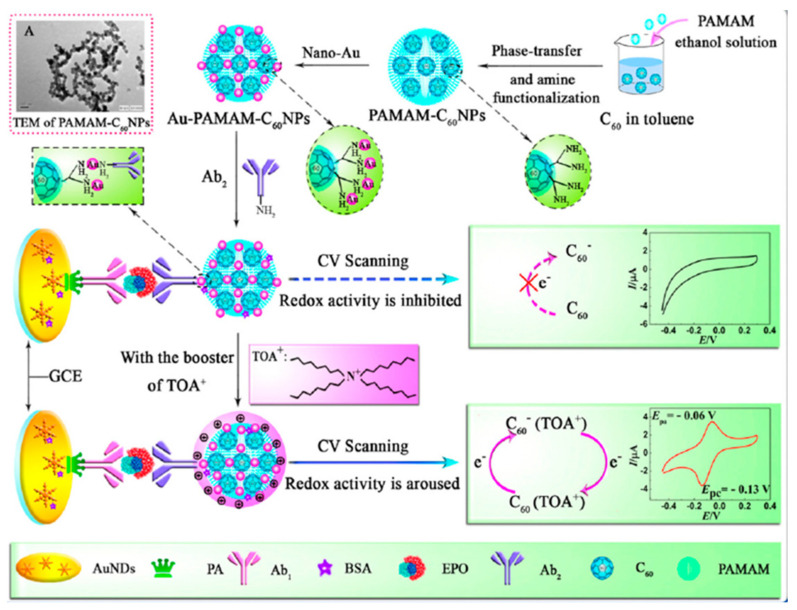
Schematic representation of the preparation of AuNPs/PAMAM/C_60_ nanocomposite used as an immunosensor for EPO detection. Reprinted from [80], copyright 2015, Elsevier.

Erythropoietin (EPO) is a key protein applied as blood-doping agent. It supports the development of novel red blood cells; hence, athletes use EPO illegally to enhance their performance by boosting oxygen delivery to the tissues. Han et al. [80] developed a sandwich-type immunosensor comprising C_60_ functionalized with PAMAM and AuNPs for EPO monitoring in human serum. As shown in Figure 9, PAMAM was dispersed in ethanol and modified with fullerene, leading to PAMAM-C60 NPs that were further coated with Au nanoparticles. An antibody was fixed onto a GCE functionalized with a tinny coating of nanodendrites and protein A. Upon incorporation of a surfactant (TOA^+^), which behaves as a supporter to stimulate the internal redox activity of C_60_, the electrochemical response was recorded by CV. The obtained immunosensor reacted linearly in the range of 0.01–80 mU/mL.

### 4.3. Sensors for Toxins

Aflatoxin B1 (AFB1) and fumonisin B1 (FB1) are among the main mycotoxins. Moreover, according to the IARC [81], AFB1 is registered as a group I carcinogen and is one of the most poisonous mycotoxins due to its ability to join DNA, thus growing the hazard of cancer in humans [82]. The FDA fixed the maximum AFB1 level at 0.3 µg mL^−1^ [83]. Over years, due to the necessity of detecting mycotoxins at extremely low concentrations, numerous biosensors have been developed. Wang and coworkers [84] prepared an SPCE incorporating G coated with polydimethylsiloxane (PDMS) for AFB1 quantification via magnetic assembly. EIS measurements were successfully applied to determine this toxin in the range of 20 to 50 ng mL^−1^, with an extremely low LOD of 0.015 ng mL^−1^. The developed method was used to determine AFB1 in nuts spiked with low amounts of standards, and very high recoveries were attained.

An electrochemical immunosensor for fast and sensitive detection of two mycotoxins, FB1 and deoxynivalenol (DON), was designed by Gunasekaran et al. [85]. A disposable SPE was used as the working electrode and was coated with a thin film of rGO, polypyrrol and AuNPs. After the best test conditions were selected, the LOD and linear range were 4.2 ppb and 0.2 to 4.5 ppm for FB1 and 8.6 ppb and 0.05 to 1 ppm for DON. The immunosensor was applied to detect these analytes in spiked corn samples, leading to good sensitivity and very few matrix interferences. Hence, it can be applied to detect contaminant mycotoxins in foodstuff and harvests.

**Figure 10:**
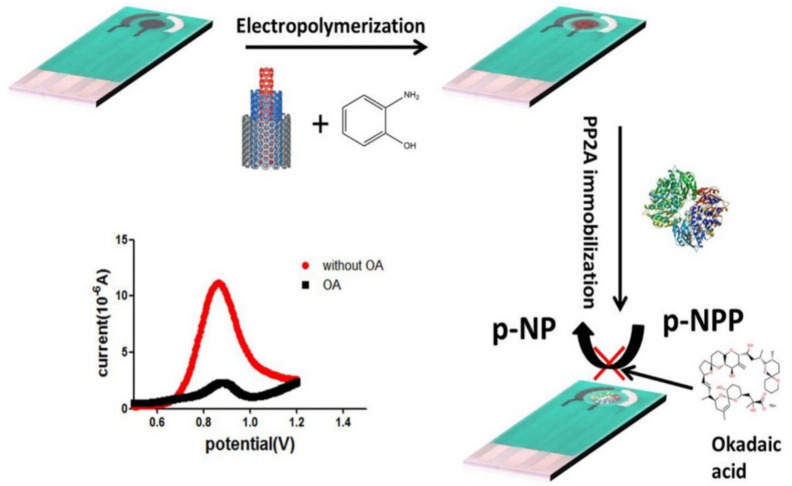
Poly-o-aminophenol (PoAP)-CNTs electrode for the development of an electrochemical biosensor for okadaic acid (OA) detection. Reprinted from [87], copyright 2016, Elsevier.

Okadaic acid (OA) is a marine toxin that gathers in the digestive glands of shellfish, dangerous for human ingestion since it blocks the active sites of protein phosphatase enzymes (PP1 and PP2A), leading to stomach complications. According to the EU Regulation [86], the concentration for human ingestion should not exceed 160 g/kg of living mollusks. An electrochemical biosensor comprising SPCEs functionalized with a CNT/poly-o-aminophenol (PoAP) nanocomposite for OA detection was designed via electropolymerization and PP2A immobilization (Figure 10). Next to OA standard incubation (or the real samples) on the modified SPCE electrode and incorporation of a phosphate derivative, OA determination was performed via DPV, within a linear range of 1–300 g L^−1^, with an LOD of 0.55 g L^−1^ [87].

Biological toxins have a crucial role in security and health areas. They are compounds fabricated by alive creatures that can provoke damaging consequences in other organisms when inhaled, ingested, or absorbed. Amongst the most toxic is botulinum neurotoxin (BoNT), which is frequently involved in intoxications and has been regarded as a biological terrorism agent due to its high toxicity and lack of antidotes [88]. Other biological terrorism agents like *Brucella* sp., *Bacillus antracis*, and *Staphylococcal enterotoxin B* can be synthesized by army or revolutionaries; hence, novel methods for fast and in situ identification of these toxins are essential in forensics [89]. In this regard, Afkami et al. [90] produced a nanocomposite coating containing AuNPs/chitosan/G for BoNT sensing. Impedance measurements were carried out in the presence of the BoNT antibody fixed on a modified GCE. BoNT was measured in serum and milk, with an LOD of 110 ng mL^−1^ and a linear range between 0.27 and 270 pg mL^−1^.

On the other hand, Cheng and coworkers [91] developed a simple, sensitive, and fast fluorescence sensor to detect CN^−^ comprising polyethylenimine (PEI)-CQDs. The surface NH_2_ moieties of the nanocomposite can take Cu^2+^ cations in a fast and sensitive way to yield cupric amine, which can quench the fluorescence of PEI-GQDs. CN^−^ inhibits that the Cu^2+^ quenches the fluorescence of PEI-GQDs; that is, cyanide anions can “turn on” the fluorescence of PEI-GQDs/Cu^2+^. This biosensor was used to detect CN^−^ with an LOD of 0.66 μM and a linear response range from 200 to 2 μM.

### 4.4. Sensors for Explosives

The detection of artifacts, particularly 2,4,6-trinitrotoluene (TNT), is of great interest around the world due to human health and security issues. In fact, these types of explosives are environmental contaminants, especially of natural waters. The design of novel fast, cheap, and consistent tests for explosive sensing both in aqueous and gas media is crucial in the field of forensics. In this regard, Shahdost-Fard and coworkers [92] developed an aptasensor for very sensitive TNT detection. A TNT/aptamer mixture was attached to AuNPs@C_60_-modified GCE via covalent bonding, and subsequent DOP polymerization was performed. After the removal of TNT, the MIP acts synergistically with the embedded aptamer to form a nanohybrid receptor (aptamer-MIP), with better recognition properties than the aptamer alone. This strategy provided a wide linearity interaval, from 0.01 fmol·L^−1^ to 1.5 µmol L^−1^, and an extremely small LOD of 3.5 amol L^−1^. This strategy may be extended to other molecules such as antibodies, proteins, and peptides.

**Figure 11:**
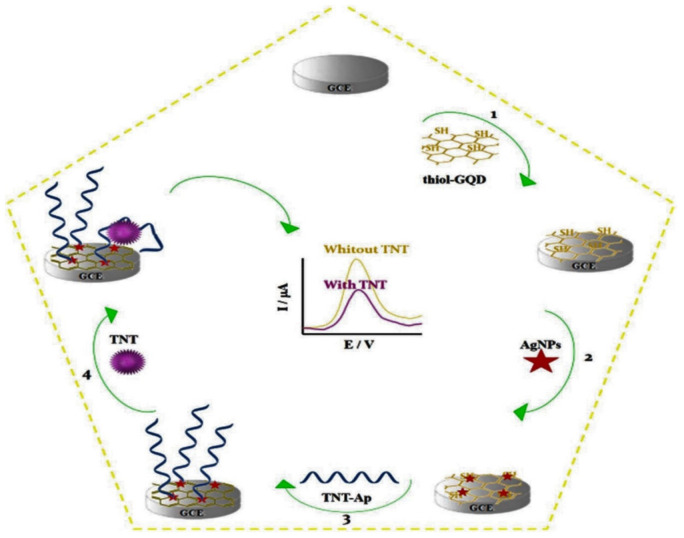
Scheme for the synthesis of an electrochemical aptasensor for quantification of TNT. Taken from [93], copyright 2019, Elsevier.

The same authors developed another TNT aptamer sensor via covalent anchoring to an AgNPs/thiol-GQD nanocomposite and used Ru as the electrochemical indicator (Figure 11) [93]. Upon binding TNT to the surface of the aptamer-modified electrode, a complex was formed that caused folding of the aptamer structure; hence, the DPV signal of Ru was reduced. This novel aptasensor was applied to quantify this explosive in water and soils with very satisfactory results, ascribed to the improved performance of the synthesized nanocomposite, the high concentration of the surface-immobilized aptasensor, and the specificity of the selected redox system.

Shi et al. [94] reported an effective strategy for recognizing TNT using G-PANI nanocomposites. For this purpose, picric acid was utilized as a template to prepare an MIP film, and the recognition points were created upon extraction of the imprinting template. The morphology of the films was investigated via SEM, and the quantification of TNT was carried out via DPV and CV. The developed approach could also be used for environmental remediation and in the field of sample separation.

Shamsipur et al. [95] also designed an electrochemical sensor for the determination of the same explosive comprising an rGO-poly(amidoamine) hybrid (ErGO-PAMAM). The fabricated electrode was examined via FTIR, EIS, SEM, AFM, and EDXS, leading to a better response than other modified electrodes based on G, with an interval of linearity from 0.05 to 1.3 ppm, and a small LOD of 0.002 ppm.

### 4.5. Sensors for Toxic Gases

Graphene functionalized with polymers has been used for sensing toxic gases since it presents a porous structure that improves the diffusion of molecules to the sensing layers. Furthermore, macromolecules can interact via H-bonding or π-π stacking with specific gases, which results in better sensitivity.

**Figure 12:**
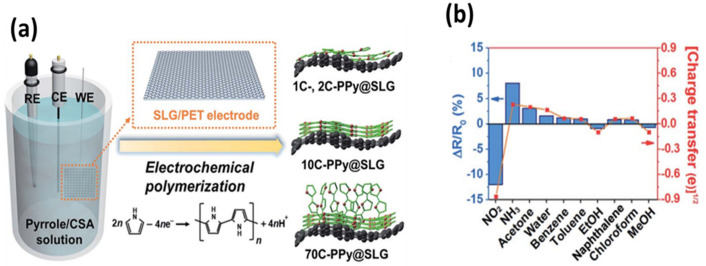
(**a**) Synthesis of polypyrrole (PPy) on a single-layer graphene/polyethylene terephthalate (SLG/PET) film using a three-electrode system and representation of the electrochemical polymerization and structure after the application of 1, 10, and 70 cycles to the nanocomposite. (**b**) Assessment of the sensor selectivity for several gases at a concentration of 1 ppm. Adapted from [96], copyright 2018, Royal Society of Chemistry.

Yoon and coworkers [96] prepared a flexible and transparent sensor that included CVD graphene functionalized with PPy. Modification was attained by electrochemical polymerization of the monomers on a single-layer graphene/polyethylene terephthalate (SLG/PET) film (Figure 12a), which were subjected to up to 70 cycles, showing outstanding cyclability. The manufactured sensor was applied to quantify several gases (Figure 12b), including NO_2_ and ammonia, and the lowest LOD values were registered for these two compounds (0.03 and 0.04 ppbs, respectively). The sensor electrical resistance dropped after contact with NO_2_, whereas it increased after contact with NH_3_. The outstanding sensing capacity of the nanocomposite was attributed to the arranged PPy/graphene structure, which improves the charge mobility.

Using an analogous tactic, NH_3_ sensors were built by PPy electropolymerization on CVD-G [97], and a better response was found compared to a sensor that lacked the polymeric layer. Very good selectivity was found when the sensors were brought in contact with 5 ppm of NH_3_ at 25 °C in the presence of CH_2_O and NO_2_ gases, which can act as interferences. The main mechanism for the superior sensitivity was believed to be the electron transfer from NH_3_ to PPy.

**Figure 13:**
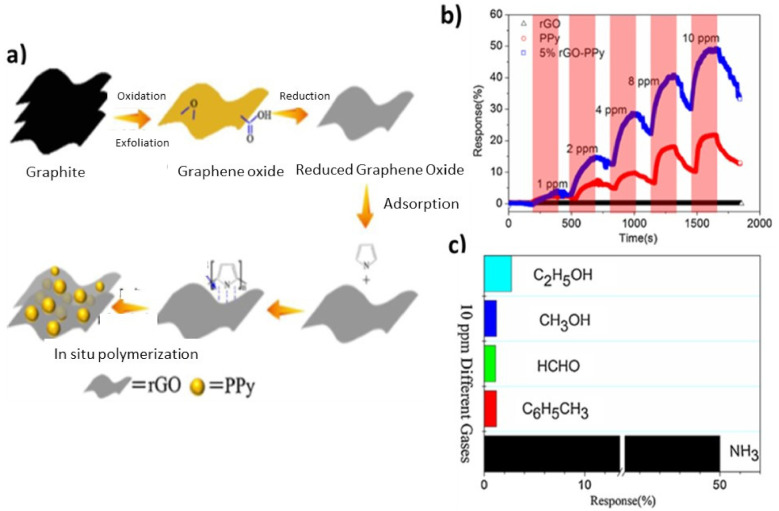
(**a**) In situ polymerization of polypyrrol onto rGO via oxidative exfoliation and reduction. (**b**) Response of rGO, neat PPy, and rGO/PPy nanocomposite to 1–10 ppm of NH_3_. (**c**) Selectivity of rGO/PPy to different gases at a concentration of 10 ppm. Adapted from [98], copyright 2017, with permission from Elsevier.

PPy/rGO hybrids were also used to develop a gas sensor for NH_3_ [98], in which PPy was anchored to rGO via an in situ chemical oxidation polymerization mechanism (Figure 13a). Raman and FTIR studies corroborated the H-bonding and π stacking interactions between the polymer and the nanomaterial, which led to increased sensor resistance. The response of rGO, neat PPy, and rGO/PPy nanocomposite versus ammonia concentrations in the range of 1–10 ppm was recorded (Figure 13b), and a linear response was found in this concentration range, showing good selectivity when alcohols or other gases such as formaldehyde were present, as depicted in Figure 13c.

Using a similar approach, Tiwari et al. [99] prepared PPy/rGO hybrids by in situ oxidative polymerization. FTIR and XRD studies indicated the existence of π-π stacking interactions between PPy and rGO g. The gas sensing efficiency was evaluated for NH_3_ concentrations in the range of 5–300 ppm, and very good sensitivity was found, ascribed to the improved adsorption capability due to the big surface area and porous nanocomposite structure. PPy and G are p-type semiconductors, whereas NH_3_ is an n-type one, and this results in modification of the resistivity of the sensor.

Lately, Tang et al. [100] prepared similar PPy/rGO ammonia sensors with a controllable thickness. GO was deposited on the electrodes and then thermally reduced, followed by electropolymerization of a thin PPy coating on the reduced GO surface. The response of the electrode was assessed for NH_3_ contents in the range of 1–5 ppm, and a linear response was observed. The detection mechanism was related to the transfer of electrons between the nanocomposite and the NH_3_ molecules. The recovery time was increased by annealing at a moderate temperature. Besides, the selectivity was assessed and compared to other gases, including HCO and H_2_, and hardly any response to these interferences was found, thereby confirming the outstanding selectivity to ammonia. Thus, synergistic effects between the polymer and the graphene derivative resulted in superior sensitivity and selectivity compared to the sensor comprising only the neat rGO.

A comparison of the most relevant carbon-based polymeric nanocomposites used in forensic analysis for the determination of different analytes, including drugs, doping substances, toxins, and explosives, along with the LOD and the linear range of the developed methos is provided in Table 1.

## 5. Conclusions

Recently, the development of novel polymer/carbon nanomaterial nanocomposites has improved forensic investigation by speeding up the forensic procedures and contributing to crime prevention and solving. Many forensic applications such as the detection of illegal drugs, doping substances, toxins, explosives, etc. have been advanced by the utilization of these novel nanocomposites. These nanocomposites comprise nanomaterials such as fullerenes, quantum dots, carbon nanotubes, and graphene and its derivatives graphene oxide and reduced graphene oxide in order to enhance the results of tracing, detection, and analysis in forensic research. Advancements have arisen from the combination of various analytical techniques such as XRD, FTIR, Raman spectroscopy, AFM, TEM, SEM, NMR, GC/MS, and LC/MS. In forensics, sample traces are generally pursued; hence, their detection is typically an awkward procedure. However, with the expanding technology in polymeric nanomaterials, on-the-spot identification and quick analysis are realized.

Carbon-based polymeric nanosensors show a better performance compared with those without carbon nanomaterials regarding their selectivity, sensitivity, and limit of detection. Moreover, they are more sustainable. However, while a lot of advancement has been attained in the arena of these multifunctional sensors, numerous queries and encounters remain. On the nanomaterial fabrication side, changes from sensor to sensor are challenging. In order to ensure reproducibility, standardized methods for carbon nanomaterial synthesis are still lacking, which are a key issue conditioning device performance. Once the nanosensors are prepared, additional characterization is required via microscopic and spectroscopic techniques in order to investigate the polymer–carbon nanomaterial interactions. Additional struggle is needed to fully investigate the sensing mechanisms and the parameters influencing the device performance. Theoretic calculations will be highly valuable in interpreting the interactions taking place between the polymer, the carbon nanomaterials and the specific analyte with a view to target certain analytes.

In this regard, the challenge is the design of new approaches to improve the sensitivity to analytes with weaker interaction. Moreover, the response time of these polymer nanocomposites has hardly been investigated compared to other parameters such as sensitivity or selectivity, yet it can be critical for numerous applications. An additional focus on investigating the response time limits and accurately rating this parameter in this type of sensor will be desirable in future research. Another issue is the investigation of potential toxicity. The toxicity of carbon nanomaterials remains unknown and should be fully investigated prior to scale up and commercialization of these nanosensors.

Amongst the foremost challenges in confirming the selectivity of these sensors is trying them in real situations. Future work should focus on this stage and provide informative data on the selectivity under real conditions. It is also expected that more efforts will be devoted to design and implement sensors that can concurrently detect several analytes. Together with properties that have already been corroborated such as mechanical flexibility, selectivity, and sensitivity, these nanosensors will likely become the next generation of monitor devices in forensic sciences.

## Figures and Tables

**Table 1:** Comparison of carbon-based polymeric nanocomposites used in forensic sciences for the determination of different analytes.

Nanocomposite	Analyte	LOD	Linear Range	Ref.
Poly C-DNA/Tween 20/GO	COC	2.45 pM	-	[57]
Nafion/SWCNT	MO	0.02 µM	0.05–1 µM 1–10 µM	[59]
Nafion/SWCNT	CO	0.05 µM	0.1–50 µM	[59]
Nafion/SWCNT	OX	85 nM	0.5–10 µM	[60]
MIP/GO/CDs NPs	OX	0.8 ng/mL	1–2000 ng/mL	[61]
PGE/CuONPs/PPy	TRA	1.0 nM	5 nM–380 µM	[65]
MIP/G/AgNPs	TRA	2.0 nM	3.5 nM–10 mM	[66]
EμPADs/GO	KET	0.001 nM	0.001–5 nM	[67]
Zr-MOF/MIP/G	KET	0.04 nM	0.1 nM–0.4 M	[68]
PET/G	MET	0.3 g/mL	-	[69]
Fe3O4@PPy/ErGO/GCE	MET	0.01 µM	0.0005–0.2 mM	[70]
MIP/Pd-G	COC	0.05 M	0.1–0.5 M	[71]
PLA/G	COC	6 µM	20–1005 µM	[72]
rGO/PANI/AuNPs	COC	0.03 nM	0.09–85 nM	[74]
G/PANI	XYL	0.06 µg/mL	0.18–5 µg/mL	[78]
C_60_/PAMAM/AuNPs	EPO	–	0.01–80 mIU/mL	[80]
SPCE/AuNPs/G/PDMS	AFB1	15 pg/mL	20 pg/mL–50 ng/mL	[84]
PPy/AuNPs/ErGO	FB1 DON	4.2 ppb 8.6 ppb	0.2–4.5 ppb 0.05–1 ppm	[85]
PoAP/CNT/SPCE	OA	0.55 g/L	1–300 g/L	[87]
AuNPs/g-Chitosan	BoNT	0.11 pg/mL	0.27–270 pg/mL	[90]
PEI-GQDs	CN-	0.66 μM	2–200 μM	[91]
AuNPs@C_60_/MIP/GCE	TNT	3.5 amol/L	0.01 fmol/L–1.5 µmol/L	[92]
ErGO-PAMAM	TNT	0.002 ppm	0.05 to 1.3 ppm	[95]
PPy/G	NO_2_NH_3_	0.03 ppb 0.04 ppb	-	[96]
PPy/G	NH_3_	5 ppm	-	[97]
PPy/rGO	NH_3_	-	1–10 ppm	[98]
PPy/rGO	NH_3_	-	5–300 ppm	[99]
PPy/rGO	NH_3_	-	1–4 ppm	[100]

COC: cocaine; MO: morphine; CO: codeine; OX: oxycodone; TRA: tramadol; KET: ketamine; MET: methamphetamine; XYL: Xylazine; EPO: Erythropoietin; AFB1: aflatoxin B1; fumonisin B1; DON: deoxynivalenol; OA: Okadaic acid; BoNT: botulinum neurotoxin; TNT: 2,4,6-trinitrotoluene.

## Data Availability

Not applicable.
